# Intravascular papillary endothelial hyperplasia (Masson’s tumor) of the parotid gland: a case report and literature review

**DOI:** 10.1097/MD.0000000000049898

**Published:** 2026-07-24

**Authors:** Adel Azar, Ahmad Alkheder, Hala Alibrahim, Adham Bader Aldeen Mohsen

**Affiliations:** a Department of Otorhinolaryngology, Al Mouwasat University Hospital, Damascus University, Damascus, Syria; b Faculty of Medicine, Damascus University, Damascus, Syria; c Faculty of Medicine, Syrian Private University, Damascus, Syria.

**Keywords:** intravascular papillary endothelial hyperplasia, IPEH, Masson’s tumor, parotid, review

## Abstract

**Background:**

Intravascular papillary endothelial hyperplasia (IPEH), or Masson’s tumor, is a rare benign vascular proliferation that uncommonly involves the parotid gland.

**Methods:**

This report details the case of a 53-year-old female who presented with an 8-year history of a right parotid mass that exhibited significant growth over 2 years, accompanied by the development of multiple bluish cutaneous lesions in the subauricular and cervical region.

**Results:**

Clinical and radiological evaluations were nonspecific. A cutaneous biopsy revealed a cavernous hemangioma. The patient underwent a superficial parotidectomy with complete facial nerve preservation. Histopathological examination of the parotid specimen confirmed a diagnosis of IPEH, characterized by papillary fronds lined by bland endothelial cells. Notably, one of the excised skin lesions also showed histological features of IPEH, alongside hemangioma.

**Conclusion:**

This case is exceptional as it represents a mixed-form IPEH arising in association with a preexisting vascular lesion and demonstrates rare multifocal involvement of both the parotid gland and the skin. It underscores the diagnostic challenge this entity poses, often mimicking other salivary gland tumors, and highlights histopathology as the diagnostic cornerstone. Complete surgical excision remains the treatment of choice and is curative. This case contributes to the limited literature on parotid IPEH and illustrates its potential for multifocal presentation in the context of underlying vascular pathology, making this the seventh documented case in the literature.

## 1. Introduction

Intravascular papillary endothelial hyperplasia (IPEH), 1st described by Pierre Masson in 1923 as “hémangioendothéliome végétant intravasculaire,” is an uncommon benign vascular lesion characterized by reactive proliferation of endothelial cells forming papillary structures within vascular spaces.^[1,2]^ Although it can occur in any blood-bearing tissue, it most frequently involves the skin and subcutaneous tissues of the head, neck, fingers, and trunk.^[3,4]^ IPEH is historically classified into 3 subtypes: a pure (primary) form arising in dilated vascular spaces; a mixed (secondary) form developing in preexisting vascular lesions such as hemangiomas; and a rare extravascular variant occurring in organized hematomas.^[3,5]^ The parotid gland represents an exceptionally rare site of involvement, with only a handful of cases documented in the literature to date.^[4,5]^ Clinical and radiological findings are often nonspecific, mimicking more common salivary gland neoplasms, thereby posing a diagnostic challenge.^[2,3]^ Histopathological examination remains the cornerstone for definitive diagnosis, revealing papillary fronds lined by bland endothelial cells in the absence of significant atypia, necrosis, or mitotic activity.^[3,4]^

In this report, we present a case of IPEH involving the parotid gland in a middle-aged female. To our knowledge, this represents the seventh documented case of parotid IPEH in the medical literature,^[2–7]^ underscoring its rarity and contributing to the evolving understanding of its clinicopathological spectrum.

## 2. Case presentation

A 53-year-old female patient presented to the Department of Otolaryngology-Head and Neck Surgery with a chief complaint of swelling below the right auricle. The swelling had been present for 8 years, remaining stable in size for 6 years. Over the past 2 years, the mass exhibited significant growth. Concurrent with this accelerated growth phase, the patient developed multiple bluish cutaneous lesions of varying sizes, predominantly involving the subauricular area, the right cervical skin, and the auricular helix. The patient denied pain, numbness, facial muscle weakness, paralysis, or systemic symptoms. Her past medical history was unremarkable.

**Figure 1.**
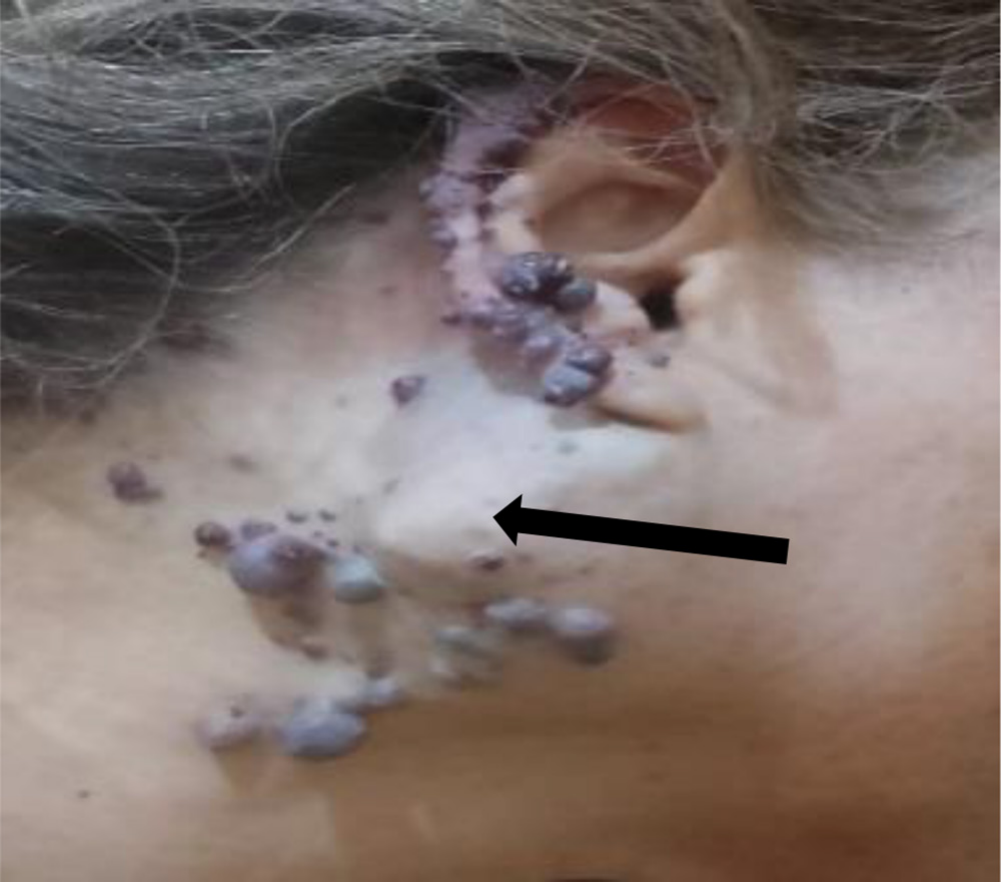
Mass below the right auricle (arrow), with multiple lesions spread over the skin.

On physical examination, a mass was noted inferior to the right auricle. Its anterior–superior border extended toward the auricle, causing slight protrusion. The mass was tender on palpation, with no signs of local fever. It was well-circumscribed, and the overlying skin was freely mobile. Multiple bluish cutaneous lesions of different sizes were observed overlying the mass, in the lateral cervical region, and on the auricular helix (Fig. 1). No lymphadenopathy was detected, and bilateral facial nerve function was intact. The remainder of the head and neck examination was within normal limits.

**Figure 2.**
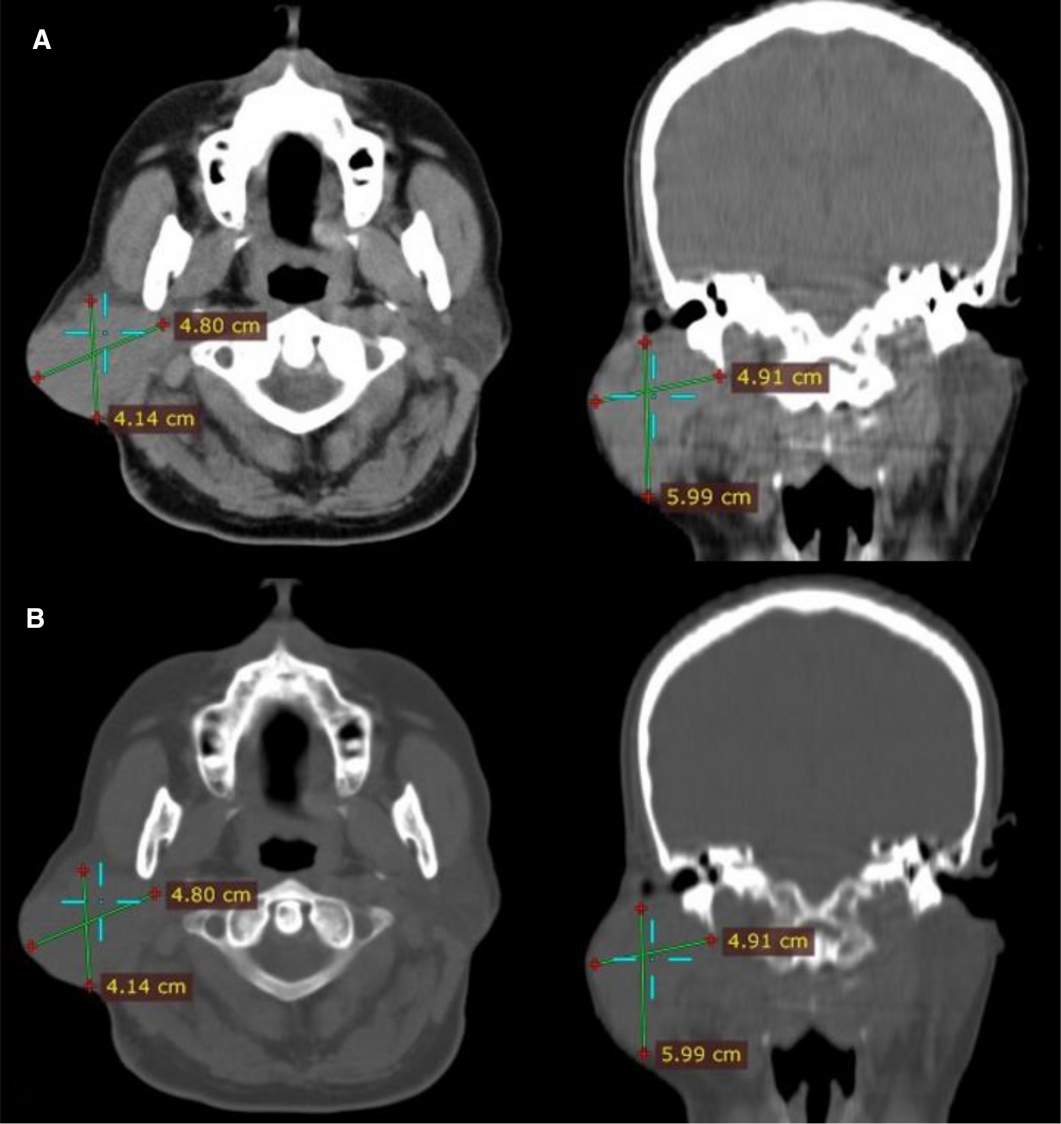
Axial and coronal CT scan revealing a homogeneous, moderately intense lesion in the superficial lobe of the right parotid gland. (A) Soft-tissue window. (B) Bone window. CT = computed tomography.

Ultrasonography demonstrated a lobulated, hyperechoic lesion in the right parotid gland, exhibiting both arterial and venous blood flow, with internal hyperechoic septations. Computed tomography revealed a well-circumscribed, round mass located in the superficial lobe of the right parotid gland, measuring 5.99 × 4.91 × 4.14 cm. The lesion showed soft-tissue attenuation, appeared relatively homogeneous on non-contrast images, and contained no calcifications or necrotic areas (Fig. 2).

**Figure 3.**
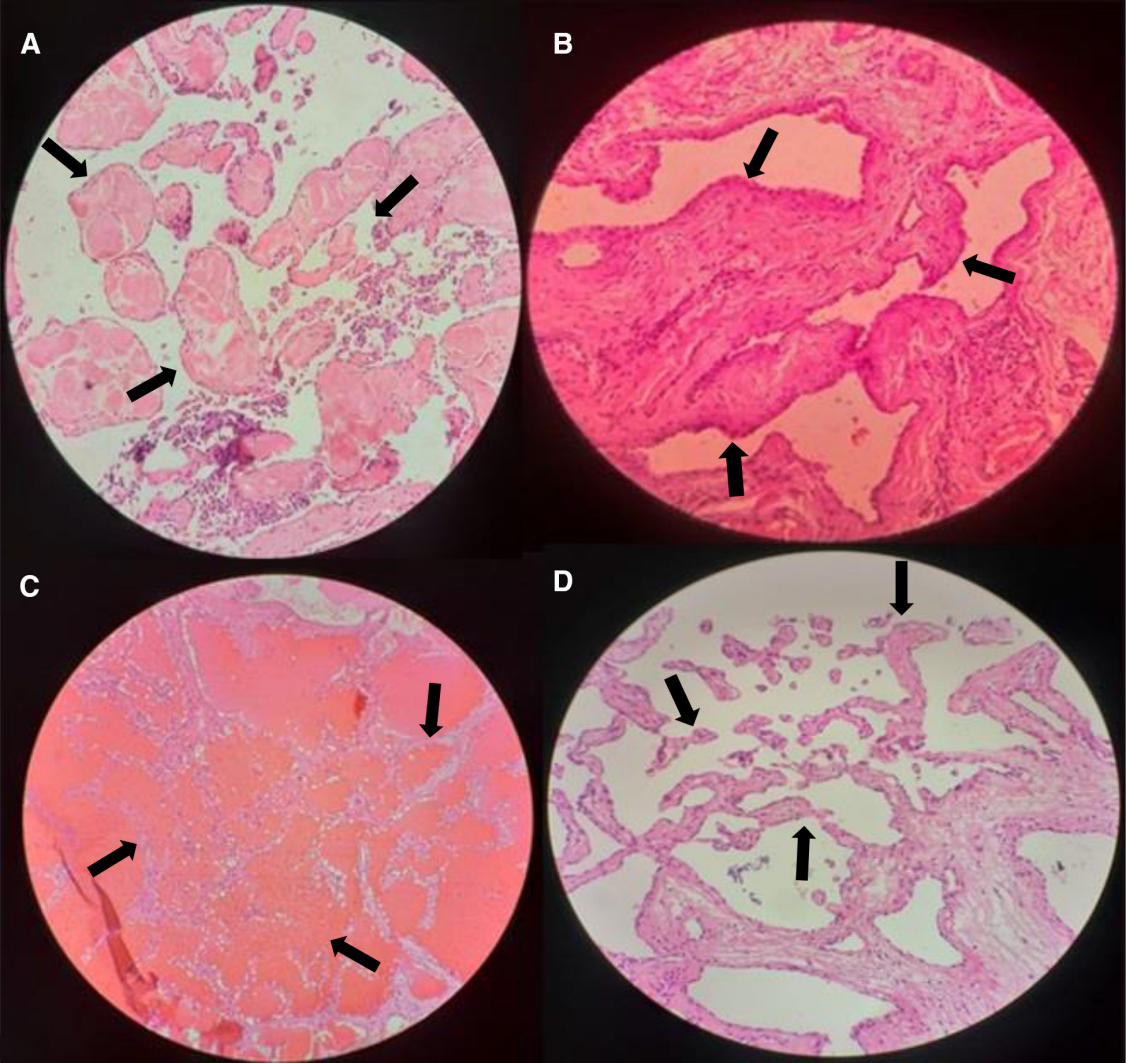
Representative histopathological images demonstrating vascular lesions. (A) Preoperative skin lesion showing features of a cavernous hemangioma, characterized by dilated vascular spaces (arrows). (B) Resected parotid mass revealing papillary endothelial hyperplasia with intravascular papillary proliferations lined by endothelial cells (arrows). (C) Excised cutaneous cavernous hemangioma demonstrating large blood-filled vascular channels separated by thin septa (arrows). (D) Resected skin lesion showing papillary endothelial hyperplasia with papillary structures projecting into the vascular lumen (arrows).

**Figure 4.**
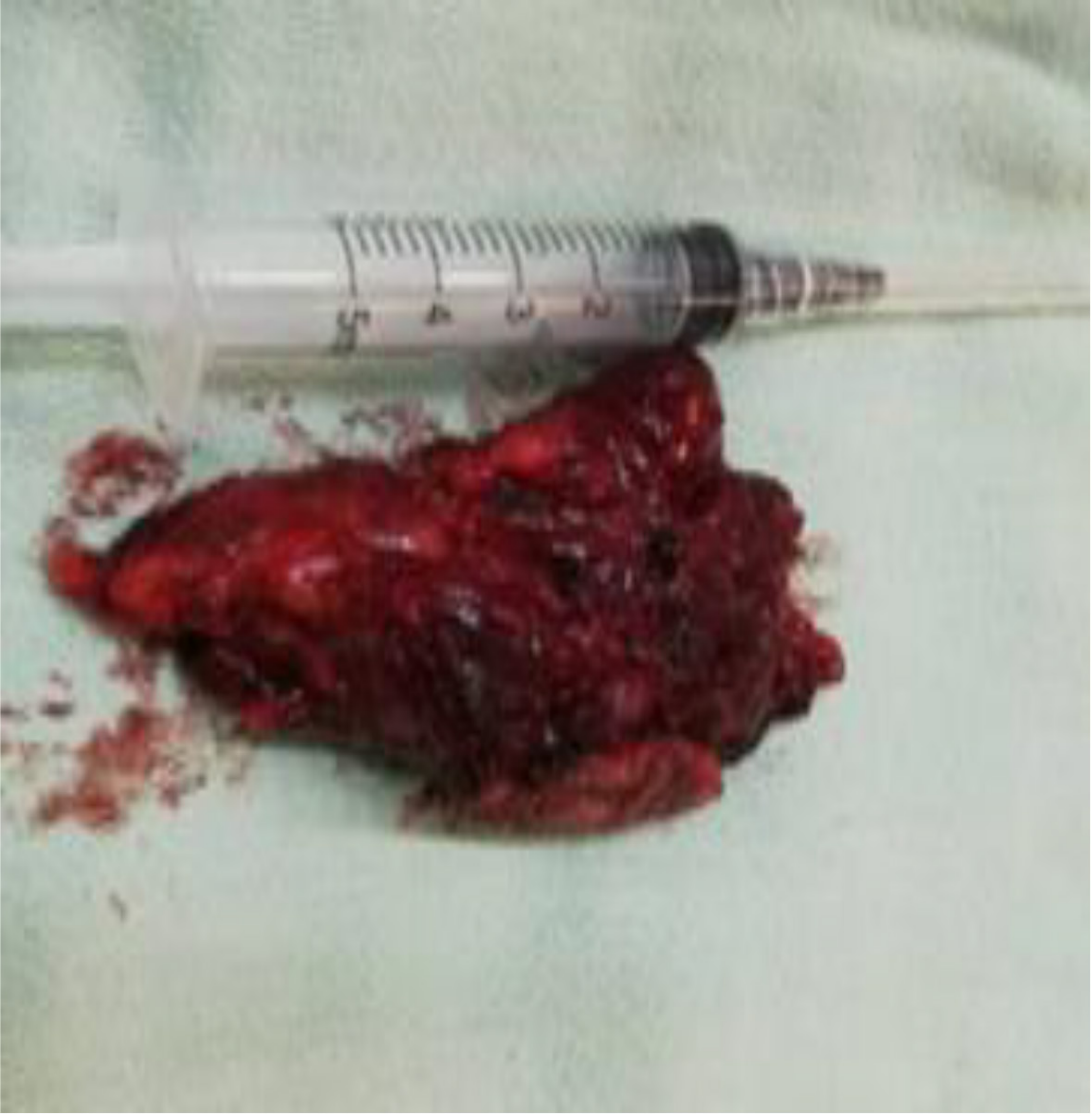
The mass excised during surgery.

A biopsy of one of the cutaneous lesions revealed a cavernous hemangioma (Fig. 3A). The patient underwent a superficial parotidectomy with preservation of all facial nerve branches (Fig. 4). The patient was discharged in very good general condition, with complete preservation of facial nerve function.

Histopathological examination of the parotid mass revealed vascular proliferation consistent with papillary endothelial hyperplasia, with no evidence of malignancy (Fig. 3B). A representative excision of 1 cutaneous lesion confirmed vascular proliferation consistent with cutaneous hemangioma (Fig. 3C). Interestingly, one of the skin lesions showed histological features of papillary endothelial hyperplasia, mirroring the findings within the parotid lesion (Fig. 3D).

## 3. Discussion

IPEH, or Masson’s tumor, is a rare, benign vascular lesion that most commonly occurs in the skin and subcutaneous tissues of the head, neck, fingers, and trunk.^[2,3,8]^ Its presentation within the parotid gland is exceedingly uncommon, with fewer than 10 cases documented in the literature to date.^[2–7]^ The present case, occurring in a 53-year-old female with a long-standing parotid mass and associated cutaneous hemangiomas, underscores the diagnostic and therapeutic challenges posed by this entity, while also contributing to the growing clinicopathological understanding of parotid IPEH.

Clinically, IPEH often presents as a slow-growing, sometimes painful mass without specific features that distinguish it from more common salivary gland neoplasms.^[2,4,5]^ In the parotid region, it may mimic pleomorphic adenoma, hemangioma, or even malignancy, both on physical exam and imaging.^[3,5,8]^ In the present case, the initial clinical and radiological findings were nonspecific, and the coexistence of cutaneous hemangiomatous lesions further complicated the preoperative assessment. This association is consistent with the “mixed” or secondary form of IPEH, which arises in the setting of preexisting vascular abnormalities such as hemangiomas.^[3,5,8]^ The presence of both intravascular (parotid) and extravascular (cutaneous) IPEH components in the same patient is highly unusual and suggests a common pathogenic mechanism, possibly related to localized vascular instability or trauma.^[3,8]^

A comparative synthesis of the 7 documented cases of parotid IPEH, including the present report, reveals instructive patterns and underscores the uniqueness of our patient’s presentation (Table 1). Demographically, there is a marked female predominance (5 out of 7 cases), with patient ages ranging from 29 to 70 years. Clinically, the most consistent feature is a slow-growing, predominantly painless parotid mass, with a symptom duration varying from 9 months to 17 years before intervention. Our case is exceptional for its unusually long history of 8 years with recent accelerated growth, and most notably, for the concomitant multifocal cutaneous involvement. Radiologically, magnetic resonance imaging and computed tomography have been the mainstays for preoperative assessment, typically revealing well-circumscribed, enhancing lesions, though none with pathognomonic features. The diagnostic challenge is evident, as initial impressions often suggested more common entities like pleomorphic adenoma or hemangioma. Histopathologically, all cases were confirmed by the characteristic papillary proliferation of bland endothelial cells. Therapeutically, complete surgical excision – via superficial, total, or partial parotidectomy – has been the unequivocal standard, with facial nerve preservation successfully achieved in all detailed reports. Preoperative embolization was utilized in 1 highly vascularized tumor. Outcomes have been uniformly excellent, with no reported recurrences in cases with follow-up data, reinforcing the benign nature of this lesion when fully resected. Our case aligns with the mixed (secondary) subtype of IPEH, arising in association with a preexisting cavernous hemangioma, a feature shared with only 1 other reported parotid case. However, the synchronous presentation of histologically confirmed IPEH in both the deep parotid parenchyma and the overlying skin represents a singular phenomenon not previously described, highlighting a potential for multifocality within a field of vascular pathology.

**Table 1:** Summary of all cases of intravascular papillary endothelial hyperplasia (Masson’s tumor) in the parotid gland reported in the literature.

Author (year)	Tumor location	Age/sex	Clinical presentation and duration	Preoperative and diagnostic work-up	Treatment	Outcome/follow-up
Corio et al. (1982)^[7]^	Parotid (side NS)	Details NS	Painless mass	Histological examination	Excision	NS
Mokhtari et al. (2011)^[6]^	Left parotid	39-yr-old male	1-yr history of painless enlargement	No imaging reported; histological diagnosis	Partial parotidectomy	NS
Carta et al. (2018)^[4]^	Right parotid	43-yr-old female	4-yr history of slow-growing, mildly painful mass (~2 cm)	Contrast-enhanced MRI	Subtotal parotidectomy with FN preservation	30 mo, symptom-free, intact FN
Mignogna et al. (2017)^[3]^	Right parotid	70-yr-old female	5-yr history of painless enlargement	MRI, 3D-CT	Extracapsular dissection	NS
Mohebbi et al. (2023)^[2]^	Right parotid	29-yr-old female	17-yr history of slowly progressive swelling	Contrast-enhanced MRI	Pre-op embolization, followed by total parotidectomy with FN preservation	Uncomplicated recovery, no FN damage
Alotaibi et al. (2025)^[5]^	Right parotid	39-yr-old female	9-mo history of painless, gradually enlarging mass	CT with contrast	Superficial parotidectomy	6 mo, no recurrence, intact FN
Present case (2025)	Right parotid	53-yr-old female	8-yr stable mass with significant growth over 2 yr; multiple bluish cutaneous lesions	US, CT; cutaneous biopsy (cavernous hemangioma)	Superficial parotidectomy with FN preservation	Uneventful recovery, intact FN; Histology confirmed mixed-form IPEH in parotid and skin

CT *=* computed tomography, FN *=* facial nerve, MRI *=* magnetic resonance imaging, NS *=* not specified, Pre-op *=* preoperative, US *=* ultrasonography.

Imaging characteristics of IPEH are variable and often overlap with those of other benign and malignant parotid tumors. On magnetic resonance imaging, IPEH may appear as a well-circumscribed, lobulated lesion with heterogeneous signal intensity and contrast enhancement due to the presence of thrombotic material and papillary vascular structures.^[2,4,5]^ In some cases, as in the present 1, the lesion may demonstrate prominent vascularity on Doppler ultrasound and contrast-enhanced computed tomography.^[5,8]^ Nevertheless, none of these features are pathognomonic, and histopathological examination remains the gold standard for diagnosis.^[3,4,8]^

Histologically, IPEH is characterized by intravascular papillary proliferations of endothelial cells surrounding hyalinized cores, in association with organizing thrombus.^[2–4,8]^ Importantly, the endothelial cells exhibit minimal atypia, and mitotic figures and necrosis are absent – features that help distinguish IPEH from angiosarcoma, a critical differential diagnosis.^[3,5,8]^ Immunohistochemically, the lesional cells typically express endothelial markers such as CD31, CD34, and ERG, confirming their vascular lineage.^[3,8,9]^ In the present case, the diagnosis was further supported by the histological identification of IPEH within both the parotid and cutaneous specimens, an exceptionally rare finding that underscores the lesion’s multifocal potential in the context of underlying vascular pathology.^[3,8]^

The pathogenesis of IPEH remains incompletely understood. Originally described by Masson as a neoplastic process, it is now widely regarded as an exaggerated, reactive endothelial proliferation occurring in response to thrombosis or vascular stasis.^[3,5,8]^ Trauma, hormonal influences, and preexisting vascular malformations have all been implicated as potential triggers.^[2,3,5]^ Recent immunohistochemical studies have also suggested a role for ferritin in the pathogenesis of IPEH, possibly through the modulation of angiogenesis and endothelial cell survival.^[3,8]^ In the present case, the long history of a stable parotid mass with recent growth and the development of cutaneous lesions may indicate progressive vascular dysregulation, although the exact mechanisms remain speculative.

Recent molecular insights continue to illuminate the pathogenesis of IPEH. Thrombus-induced hypoxia stands out as a critical initiator, with hypoxia-inducible factor-1 and vascular endothelial growth factor prominently expressed in the cellular papillae during thrombus remodeling to drive endothelial proliferation and angiogenesis.^[10]^ Intussusceptive angiogenesis further contributes by enabling vessel splitting through transluminal pillars, which helps generate the papillary architecture characteristic of Masson’s tumor.^[11]^ These observations align with the prevailing understanding of IPEH as a reactive endothelial response rather than a neoplastic process, representing an exaggerated reparative reaction to organized thrombus.^[12]^

Treatment of parotid IPEH consists of complete surgical excision, which is both diagnostic and curative.^[2,4,5]^ Superficial or total parotidectomy with facial nerve preservation is the approach of choice, depending on the size and location of the lesion.^[4,5]^ Preoperative embolization may be considered in highly vascularized tumors to reduce intraoperative bleeding risk.^[2]^ In the present case, superficial parotidectomy was successfully performed with preservation of all facial nerve branches and no evidence of recurrence, consistent with the excellent prognosis typically associated with completely excised IPEH.^[2,4,5]^

IPEH of the parotid gland is a rare and diagnostically challenging lesion that may mimic both benign and malignant salivary gland tumors. Clinicians and pathologists should maintain a high index of suspicion, particularly in cases with associated cutaneous vascular lesions or suggestive imaging findings. Complete surgical excision is the treatment of choice and is associated with an excellent prognosis. Further reporting of such cases will help refine diagnostic criteria and improve our understanding of the pathogenesis and natural history of this unusual entity.

### 3.1. Limitations

This study has certain inherent limitations, primarily stemming from its design as a single case report. While it provides a detailed clinicopathological account of a rare entity, the findings and observations cannot be statistically generalized. The management and diagnostic pathway described reflect a specific clinical context at a single institution. Furthermore, while the diagnosis was definitively established via classic histomorphological features on hematoxylin and eosin staining, immunohistochemical analysis was not performed, which may be considered a diagnostic limitation in cases where endothelial markers are preferred for additional confirmation. Additionally, the literature review, though comprehensive, is susceptible to the publication bias inherent in reporting rare cases. Long-term follow-up data beyond the immediate postoperative period is not available for all historically reported cases, including our own, which limits the assessment of very late recurrence, although IPEH is considered benign with an excellent prognosis after complete excision.

## 4. Conclusion

This rare case of IPEH involving the parotid gland highlights the diagnostic challenges posed by its nonspecific clinical and radiological presentation. The coexistence of cutaneous hemangiomas and the identification of IPEH foci in both parotid and skin specimens support a mixed subtype arising from preexisting vascular lesions. Complete surgical excision remains the cornerstone of management, offering both diagnostic confirmation and cure. This report reinforces the importance of histopathological examination in distinguishing IPEH from malignant vascular neoplasms and contributes to the sparse literature on parotid IPEH, underscoring its benign yet potentially multifocal nature.

## Author contributions

**Conceptualization:** Adel Azar, Ahmad Alkheder.

**Data curation:** Hala Alibrahim.

**Methodology:** Adel Azar, Ahmad Alkheder, Hala Alibrahim.

**Project administration:** Adham Bader Aldeen Mohsen.

**Supervision:** Adham Bader Aldeen Mohsen.

**Validation:** Adel Azar, Ahmad Alkheder, Hala Alibrahim, Adham Bader Aldeen Mohsen.

**Visualization:** Adel Azar, Ahmad Alkheder.

**Writing – original draft:** Adel Azar, Ahmad Alkheder, Hala Alibrahim.

**Writing – review & editing:** Adel Azar, Ahmad Alkheder, Adham Bader Aldeen Mohsen.
